# What Psychiatry Means to Me

**DOI:** 10.4103/0973-1229.32162

**Published:** 2007

**Authors:** Helen Herrman

**Affiliations:** *Director, World Health Organization Collaborating Centre for Research and Training in Mental Health, University of Melbourne, Australia. Secretary for Publications, World Psychiatric Association, E-mail: h.herrman@unimelb.edu.au*

**Keywords:** *Public health*, *social medicine*, *health promotion*, *research dissemination*, *economic development*

## Abstract

Moving in early career from public health physician to psychiatrist gives me a public health view of psychiatry and an interest in pursuing the goals of widening access to community-based services for people with mental disorders and promoting mental health in communities. Training in social medicine in the UK and psychiatry in Australia lead to studies of homelessness in people living with psychotic disorders, the health of family caregivers, assessing quality of life and mental health promotion.

Work with the World Health Organization (WHO) in the Western Pacific Region and the World Psychiatric Association (WPA) worldwide has given me opportunities to work with psychiatrists, mental health workers, service users and others in governments and non-government organisations implementing the recommendations of the World Health Report 2001 in countries with limited resources. My work as WPA Secretary for Publications seeks to improve information exchange in countries irrespective of their wealth. This is an exciting time to be working in a global village with technical capacity to reach into its furthest corners. Psychiatrists supported by WPA can help ensure that vulnerable people and communities and people living with mental disorders are well served in this new environment and no longer left out and left behind.

## Introduction

Mental health is inseparable from health and psychiatry is integral to public health. This is the heart of what psychiatry means to me. Beginning professional life as a public health physician shapes my perceptions and gives a peculiar twist to the meaning of psychiatry. My professional journey begins with public health, includes two decades as a clinical and academic psychiatrist with responsibilities for the development of community-based services and continues as a psychiatrist with an interest in mental health and development.

## Social Medicine and Mental Health

Along the way I have been fortunate in encounters with people. Dame Rosemary Rue was regional medical officer in the Oxford Regional Health Authority (UK) in the 1970s when I was a young postgraduate student and then registrar in community medicine in Oxford. As an honorary fellow of the Royal College of Psychiatrists and an astute administrator she discerned a latent interest and proposed that I gain clinical experience in the National Health Service (NHS) at the Warneford Hospital. I had studied medicine and had early clinical experience in my home city of Melbourne in Australia. Hence I began clinical psychiatry as registrar in the day hospital at the Warneford and there encountered a long-term colleague Sidney Bloch. I obtained fellowship of the Royal Colleges of Physicians Faculty of (now) Public Health and later MD, with a dissertation on the topic of schizophrenia and physical illness based on the Oxford Record Linkage Study. Dr John Baldwin guided the work, along with David Christie on my return to Melbourne. I still recall the handwritten letter from Michael Shepherd accepting the resulting paper for Psychological Medicine (Herrman, 1983) and including a few words of encouragement. I got to know him when he made a visit to Melbourne late in his life. He recognised that experience as registrar in community medicine in Oxford and concurrently fellow in the Department of Social Medicine (later Department of Epidemiology) at Oxford had given me the privilege of encountering the legacy of John Ryle, appointed chair of the newly created Institute of Social Medicine at Oxford in 1943.

Pemberton (Pemberton, 1998) reflects on two streams of thought that came together to create social medicine in the United Kingdom. The first was the realization by doctors in clinical medicine in the 1930s that much more attention should be paid to the patient's environment when considering causation, treatment, aftercare and prevention. John Ryle recognised the injustice of the inequalities in health caused so largely by poverty. He and other doctors began to ask questions such as why did this particular patient become ill at this time and in this place? This thinking profoundly affected the practice of clinical medicine in the UK and in Australia in later decades. The second stream of thought (Pemberton, 1998) was the development of epidemiological research for investigating the causes of noncommunicable disease, especially cancer and heart disease. This stream was strongly represented by a group of epidemiologists working at the London School of Hygiene in the years immediately after the second world war, some of whom lead by Sir Richard Doll later moved to Oxford. Subsequent developments in epidemiology include the great expansion in health service research and the acceptance of the need for randomized clinical trials of new treatments. Originally published in 1972, Archie Cochrane's classic text (Cochrane, 1972; reprinted 1989, 1999) has had a profound influence on the practice of medicine and on the evaluation of medical interventions. The influential book from Geoffrey Rose (Rose, 1992) was published later, in 1992, but David Christie introduced me to his ideas before this. Rose contrasts the individual high-risk and population-based strategies of prevention and offers the concept of prevention as a matter for populations and not just for individuals. He illustrates the application of his ideas to alcoholism and depression as well as heart disease and road accidents (Rose, 1993). Later on in Melbourne I had the privilege of encountering Jeremy Anderson, who had worked with Geoffrey Rose to consider population approaches to prevention of depression.

## Psychiatry and Public Health

With my head full of social medicine and an interest in psychiatry I returned to Melbourne and gravitated to psychiatry. At this stage I was especially grateful to Rosemary Rue for her inspired notion and scheme for part-time specialty training in Oxford, which later spread to other parts of the NHS. One way and another I was able to work part-time over seven years while my two sons were babies, in Oxford and then by seeking out ways to do it with the help of flexible colleagues in Melbourne. Otherwise I may not have ventured to begin training in psychiatry at Parkville Centre and Royal Park Hospital in Melbourne in the 1980s. There I encountered the teaching and stories of Richard Ball whom I later succeeded as Professor and Director of Psychiatry at St Vincent's Hospital and the University of Melbourne.

Working at Royal Park Hospital where we had been taught by John Cade as Monash University medical students, I found that young people were being discharged after relatively brief hospital stay, jobless, to live in rooming houses. They soon returned, even more demoralised. These experiences along with seeing the inspired work of clinicians of the various disciplines in those times, influenced me to turn to social medicine or public health, in psychiatry. With colleagues at Royal Park we began investigations aiming to draw attention to homelessness in people living with psychotic disorders (Herrman, 1989) and the problem of people with mental disorders in prisons (Herrman, McGorry *et al.*, 1991; Herrman, Mills *et al.*, 1994). At that time Pat McGorry and I were awarded a grant from the National Health and Medical Research Council, to follow a cohort of young people with early onset psychotic disorders (Henry, Harris *et al.,* 1997).

## Psychiatry and Mental Health

Then followed studies on the mental health and quality of life of family caregivers (Eastwood 1991; Szmukler, Herrman *et al.*, 1996). The Victorian Health Health Promotion Foundation (VicHealth) funded these studies. I later served for nearly a decade on the Foundation's Board of Trustees chaired successively by Prof Sir Gustav Nossal and Prof John Funder. The first Chief Executive Officer was Rhonda Galbally and the second Prof Rob Moodie, both of whom were wonderful colleagues from different backgrounds and champions of mental health promotion. Later Rob Moodie and I joined Shekhar Saxena from the Department of Mental Health and Substance Abuse in WHO Geneva to edit a book on the evidence base for mental health promotion (Herrman, 2005). This remains an area of interest to work jointly on models for mental health promotion in poorly resourced countries.

Prof Norman Sartorius asked me if the work on family caregivers would extend to an interest in assessing quality of life and here began a career-long connection with the World Health Organization (WHO) and interest in quality of life assessment (WHOQOL Group, 1998; Herrman, Hawthorne *et al.,* 2002). The subjective assessment of quality of life and disability (Chopra 2004) supports the need for people living with psychotic disorders to be partners in their own care and recovery. Likewise clinicians need to work with families and consumers as participants in service planning and management (Herrman, Trauer *et al.,* 2002). The survey of psychotic disorders in Australia (Jablensky, McGrath *et al.,* 2000), studies related to that with good colleagues Carol Harvey and Oye Gureje (Gureje, Herrman *et al.,* 2002) and studies of depression in primary care (Herrman, Patrick *et al.,* 2002) confirm the value of social medicine in psychiatry. Studies of this type support advocacy and service planning and advance understanding of the illnesses.

## Promoting Mental Health and Public Health Psychiatry

A public health view of mental health is now emerging more strongly than in the recent past with the understanding that mental health for individuals and communities is the product of many interacting factors. As enshrined in the WHO definition of health, mental health and mental illness are inseparable from health and illness in general and closely related to behaviour. The links in all parts of the world between mental illness and poverty and social disruption are clear. Improving mental health consequently depends on a range of actions at several levels in any country (Cooper, 1993; Desjarlais, 1995; World Health Organization, 2001). Research has shown that mental health can be affected by non-health policies and practices, for example in housing, education and child care. Improvements in mental health are associated with improved health and productivity. Despite uncertainties and gaps in the evidence, we know enough about the links between social experience and mental health to make a compelling case to apply and evaluate locally appropriate policy and practice interventions to promote mental health (Herrman, 2005).

I have been keen to support the work of the World Psychiatric Association (WPA) in considering how the profession of psychiatry can contribute to the improvement of public health in countries, whether poorly resourced or wealthy (Herrman, 2005). Psychiatrists can have an influence on public policies affecting health care, as well as other sectors such as education, commerce, employment, housing, child and family welfare and justice. Psychiatrists have a direct influence on treatment services and standards of treatment, although often limited by lack of resources. Along with patients and their families and mental health and health care professionals, they have much to gain from the growing integration of mental health with health care.

## The WHO and National Mental Health Policies and Plans

The WHO Western Pacific Region Office (WPRO) published its Regional Strategy for Mental Health in 2002 (http://www.wpro.who.int/publications/pub_9290610077.htm). I was privileged to work as acting regional adviser in mental health for WPRO at the time of this policy development. The Western Pacific Region covers 37 countries and territories in Asia and the Pacific, stretching from China and Mongolia in the north, to Australia and New Zealand in the south and west across the Pacific countries to French Polynesia. As I joined in 2001, colleagues at WPRO were working hard to co-ordinate global, regional and national events for World Health Day 2001. I had the opportunity to participate in the launch of the World Health Report and collaborate in global projects like Project ATLAS and the Global Campaign on Epilepsy and to seek the advice of Norman Sartorius in much of this work, including the development of the regional strategy for mental health. The experience of working with colleagues, consultants and advisers in Manila, in Geneva and in countries around the region, was a highlight of this time. It reinforced for me the importance of psychiatrists working effectively with governments, professions, patients and a range of community sectors. International organisations, including WHO and other intergovernmental UN organisations and non-governmental organisations such as WPA and the WFHM, have an invaluable role as partners with national and local groups for advocacy and advice.

The regional strategy proposes two directions endorsed by all countries in the region and consistent with the recommendations of the World Health Report 2001. The first is an intersectoral approach to mental health promotion and the prevention and treatment of illness. The second is the integration of treatment for mental disorders into general health services and a more informed understanding of mental health in the wider community. In all countries only a minority of people living with treatable mental illnesses obtain access to effective treatments and prevention. This is because the stigma of mental illness discourages people from seeking treatment and because services are scarce and poorly distributed in many countries. Integrating mental health promotion into health promotion will also require a shift in thinking and community values.

The regional strategy and the World Health Report 2001 recommend the development and review of national policies on mental health. Much time has been devoted to identifying the deficits in health service provision especially in low and middle income countries. The WHO reports emphasise the value of identifying and supporting the existing foundations of mental health and mental health care in local communities, to which less attention has been devoted. Examples include support for consumers and families and including them in treatment and policy-making; coordinating the range of governmental and nongovernmental agencies providing services and assistance, especially in low-income countries after wars and disasters; developing mutual understanding and a system of referral between traditional and modern medicine; support for the health workforce in countries and their augmentation as appropriate with trained community workers; attention to the psychosocial aspects of health care, up to now honoured more in the breach than observance; and encouraging the development of a research culture and capacity. Improvements in mental health are more likely when scientific evidence is produced locally, yet reliable information is lacking in many countries, especially low income countries.

## WPA Publications

Having recognised the value of WPA's work, I was pleased to be elected to the the position of Secretary for Publications. The timely and effective dissemination of research results is essential for high standards and innovation in research and clinical services. Often in low- and middle-income countries only the free abstracts are available to clinicians. The abstracts tend to be short, poorly written and sometimes they are misleading (Short, 2007). A 10/90 divide in the publication of internationally accessible mental health literature is also evident and remains unchanged. Recent studies reveal that over 90% articles in high impact journals come from richer countries (Patel, 2001; Saxena *et al.*, 2006; Patel, 2007; White, 2007). A joint statement from editors of scientific journals publishing mental health research and the WHO offered some steps to correct these imbalances (World Health Organization, 2004; Tyrer, 2005). The WPA publications programme is committed to help improve information exchange between psychiatrists, scientists, other professionals, policymakers, politicians, service users and the general public irrespective of their country of residence and its wealth. The world of publishing is undergoing radical change. WPA will aim to ensure through partnerships with publishers, WHO and other organisations that psychiatrists and their patients in all countries obtain the benefits of new ways to exchange scientific knowledge.

## Conclusions

Psychiatrists and their national associations can make vital contributions to public health through action and advocacy. The psychiatrist has a unique perspective and authority based on understanding the multiple personal and environmental contributions to mental health and illness, at individual and population levels; and the consequences in terms of health, productivity and quality of life.

### Take Home Message

Psychiatrists and the profession of psychiatry can contribute to the improvement of public health in all countries irrespective of wealth. Taking the lead from heart health, improving mental health is the result of effective partnerships between various groups and organisations interested in the mental health of individuals and populations in a country.

## Questions That this Paper Raises

- How can psychiatry as a profession participate most actively in the improvement of public health?
- Should psychiatrists take an active interest in improving positive mental health as well as preventing and treating mental illnesses?
- How can psychiatrists partner most effectively with other groups interested in the mental health of individuals, such as service users and their families, community workers and leaders, other health workers?
- How can psychiatrists partner most effectively with other groups interested in population mental health, such as community leaders, government decision-makers and politicians?

## About the Author

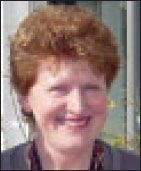



Helen Herrman is Professor in Public Health and Psychiatry at the Australian International Health Institute, University of Melbourne. From 1992 to 2005 she was Professor and Director of Psychiatry in St. Vincent's Mental Health Service Melbourne. She is Director of the World Health Organization (WHO) Collaborating Centre for research and training in mental health at the University of Melbourne and St Vincent's Health. For a year in 2001-2002 she was acting regional adviser in mental health for the WHO's Western Pacific Region. She is Secretary for Publications and member of the Executive Committee of the World Psychiatric Association and Vice-President of the International Federation of Psychiatric Epidemiology.

## References

[1] Chopra PK, Couper JW, Herrman H (2004). The assessment of patients with long-term psychotic disorders: Application of the WHO Disability Assessment Schedule II. Aust N Z J Psychiatry.

[2] Cochrane AL (1972). Effectiveness and Efficiency.

[3] Cooper B (1993). Single spies and battalions: The clinical epidemiology of mental disorders. Psychol Med.

[4] Desjarlais R, Eisenberg L, Good B, Kleinman A (1995). World Mental Health: Problems and priorities in low-income countries.

[5] Eastwood MR, Herrman HE, Singh BS (1991). Who are these informal carers. Lancet.

[6] Gureje O, Herrman H, Harvey C, Morgan V, Jablensky A (2002). The Australian National Survey of Psychotic Disorders: Profile of psychosocial disability and its risk factors. Psychol Med.

[7] Henry LP, Harris MG, Amminger GP, Yuen HP, Harrigan SM, Purcell R (2007). The EPPIC Long Term Follow-up Study of First Episode Psychosis: Methodology and Baseline Characteristics. Early Intervent Psychiatr.

[8] Herrman H (2005). Public policy and psychiatry, Advances in Psychiatry.

[9] Herrman HE, Baldwin JA, Christie D (1983). A record linkage study of mortality and general hospital discharge in patients diagnosed as schizophrenic. Psychol Med.

[10] Herrman H, Hawthorne G, Thomas R (2002). Quality of life assessment in people living with psychosis. Soc Psychiatr Psychiatric Epidemiol.

[11] Herrman H, McGorry P, Bennett P, van Riel R, Singh B (1989). Prevalence of severe mental disorders in disaffiliated and homeless people in inner Melbourne. Am J Psychiatry.

[12] Herrman H, McGorry P, Mills J, Singh B (1991). Hidden severe psychiatric morbidity in sentenced prisoners: An Australian study. Am J Psychiatry.

[13] Herrman H, Mills J, Doidge G, McGorry P, Singh B (1994). The use of psychiatric services before imprisonment: A survey and case register linkage of sentenced prisoners in Melbourne. Psychol Med.

[14] Herrman H, Patrick DL, Diehr P, Martin ML, Fleck M, Simon GE (2002). Longitudinal investigation of depression outcomes in primary care in six countries: The LIDO study Functional status, health service use and treatment of people with depressive symptoms. Psychol Med.

[15] Herrman H, Saxena S, Moodie R (2005). Promoting Mental Health: Concepts, Emerging Evidence and Practice.

[16] Herrman H, Trauer T, Warnock J, Professional Liaison Committee (Australia) Project Team (2002). The roles and relationships of psychiatrists and other service providers in mental health services. Aust NZJ Psychiatry.

[17] Jablensky A, McGrath J, Herrman H, Castle D, Gureje O, Evans M (2000). Psychotic disorders in urban areas: An overview of the Study on Low Prevalence Disorders. Aust NZJ Psychiatry.

[18] Patel V, Kim RI (2007). Contribution of low- and middle-income countries to research published in leading general psychiatry journals, 2002-2004. Br J Psychiatry.

[19] Patel V, Sumathipala A (2001). International representation in psychiatric literature: Survey of six leading journals. Br J Psychiatry.

[20] Pemberton J (1998). Social medicine comes on the scene in the United Kingdom, 1936-1960. J Public Health.

[21] Rose G (1992). The Strategy of Preventive Medicine.

[22] Rose G (1993). Mental disorder and the strategies of prevention. Psychol Med.

[23] Saxena S, Paraje G, Sharan P, Karan G, Sadana R (2006). The 10/90 divide in mental health research: Trends over a 10-year period, Br J Psychiatry. Br J Psychiatry.

[24] Short R (2007). Peer review will become “the job of the many, not the select few”. BMJ.

[25] Szmukler GI, Herrman H, Colusa S, Benson A, Bloch S (1996). A controlled trial of a counselling intervention for caregivers of relatives with schizophrenia. Soc Psychiatry Psychiatry Epidemiol.

[26] Tyrer P (2005). Combating editorial racism in psychiatric publications. Br J Psychiatry.

[27] White J, Patel V, Herrman H (2007). Australian and New Zealand contribution to international mental health: A survey of published research. World Psychiatry.

[28] World Health Organization (1998). Quality of Life Assessment (WHOQOL): Development and general psychometric properties. Soc Sci Med.

[29] World Health Organization (2001). Mental Health: New Understanding, New Hope.

[30] World Health Organization (2004). Galvanizing mental health research in low- and middle-income countries: Role of scientific journals. Bull World Health Organ.

